# Subjective cognitive decline, anxiety symptoms, and the risk of mild cognitive impairment and dementia

**DOI:** 10.1186/s13195-020-00673-8

**Published:** 2020-09-11

**Authors:** Tau Ming Liew

**Affiliations:** 1 Department of Psychiatry, Singapore General Hospital, Outram Road, Singapore, 169608 Singapore; 2 Saw Swee Hock School of Public Health, National University of Singapore, Singapore, Singapore

**Keywords:** Subjective memory complaints, Anxiety, Cox regression, Longitudinal study, Neurocognitive disorders

## Abstract

**Background:**

Subjective cognitive decline (SCD) and anxiety symptoms both predict neurocognitive disorders, but the two correlate strongly with each other. It is unclear whether they reflect two independent disease processes in the development of neurocognitive disorders and hence deserve separate attention. This cohort study examined whether SCD and anxiety symptoms demonstrate independent risks of mild cognitive disorder and dementia (MCI/dementia).

**Methods:**

The study included 14,066 participants aged ≥ 50 years and diagnosed with normal cognition at baseline, recruited from Alzheimer’s Disease Centers across the USA. The participants were evaluated for SCD and anxiety symptoms at baseline and followed up almost annually for incident MCI/dementia (median follow-up 4.5 years; interquartile range 2.2–7.7 years). SCD and anxiety symptoms were included in Cox regression to investigate their independent risks of MCI/dementia.

**Results:**

SCD and anxiety symptoms demonstrated independent risks of MCI/dementia, with HR 1.9 (95% CI 1.7–2.1) and 1.3 (95% CI 1.2–1.5), respectively. Co-occurring SCD and anxiety symptoms demonstrated the highest risk (HR 2.4, 95% CI 1.9–2.9)—participants in this group had a 25% probability of developing MCI/dementia by 3.1 years (95% 2.4–3.7), compared to 8.2 years among those without SCD or anxiety (95% CI 7.9–8.6). The results remained robust even in the sensitivity analyses that took into account symptom severity and consistency of symptoms in the first 2 annual visits.

**Conclusions:**

The findings suggest that clinicians should not dismiss one over the other when patients present with both SCD and anxiety and that both constructs may potentially be useful to identify high-risk populations for preventive interventions and trials. The findings also point to the need for further research to clarify on the neurobiological distinctions between SCD and anxiety symptoms, which may potentially enrich our understanding on the pathogenesis of neurocognitive disorders.

## Introduction

Subjective cognitive decline (SCD) refers to the *subjective* perception of a decline in cognition (typically in the memory domain) among individuals with *normal cognition* (that is, in the absence of objective cognitive deficits) [1–3]. It is increasingly common at the older ages [4]—with the literature reporting a prevalence of 50–60% among community-dwelling older persons [5, 6]—and is known to predict subsequent development of neurocognitive disorders [1, 7]. In recent years, SCD has been suggested to be useful in the diagnosis of prodromal neurocognitive disorders [1, 7], with the 2018 National Institute on Aging–Alzheimer’s Association (NIA-AA) research criteria for Alzheimer’s disease [8] incorporating SCD as a transition phase between normal cognition and early neurocognitive disorders.

**Fig. 1:**
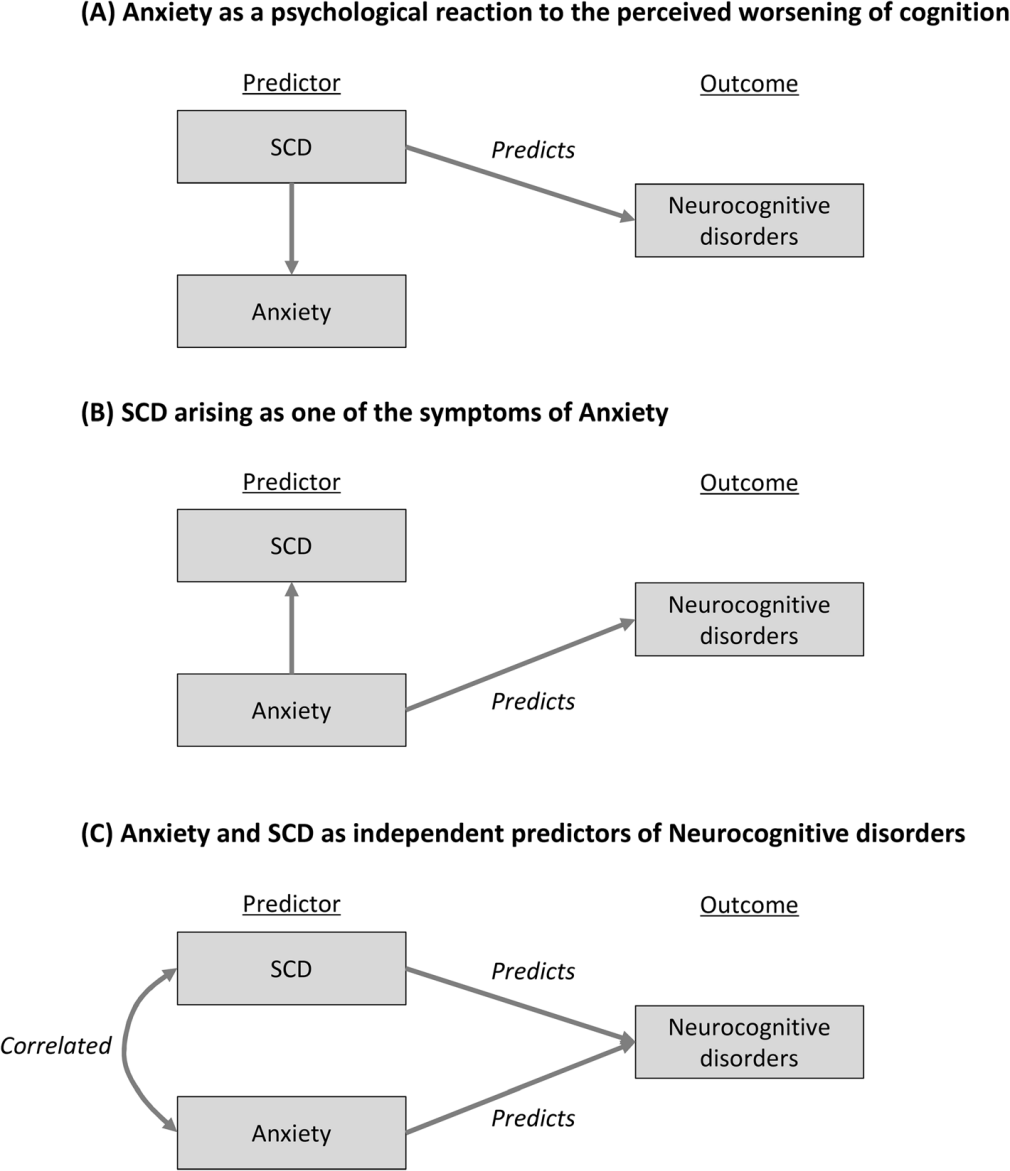
A simplified diagram to illustrate the three plausible relationships among anxiety, subjective cognitive decline (SCD), and incident neurocognitive disorders (**a**–**c**)

In the literature, SCD has been shown to correlate strongly with anxiety symptoms, with older persons often reporting both SCD and anxiety symptoms concurrently [2]. This has led to the uncertainty on whether it is SCD, or its correlated anxiety symptoms, that predicts the subsequent development of neurocognitive disorders. This is especially pertinent given that anxiety has consistently been identified as a key predictor of neurocognitive disorders in several meta-analyses [9–11]. Hypothetically, there can be at least three plausible explanations to the relationships among SCD, anxiety, and incident neurocognitive disorders, as illustrated in Fig. 1. First, SCD could be an early manifestation of neurocognitive disorders, with anxiety as a psychological reaction when a person becomes increasingly concerned about his perceived worsening of cognition (Fig. 1a). Second, anxiety could be an early manifestation of neurocognitive disorders, with worries about one’s cognitive function (SCD) as one of the many symptoms of anxiety (Fig. 1b). Third, both SCD and anxiety could also be independent predictors of neurocognitive disorders and reflect two independent disease processes that deserve separate attention (Fig. 1c).

This unaddressed gap in the literature can have critical implications to the research and clinical practice. If anxiety is merely the consequence of SCD (Fig. 1a), or vice versa (Fig. 1b), it will compel researchers and clinicians to focus on one over the other. On the other hand, if SCD and anxiety are both independent predictors of neurocognitive disorders (Fig. 1c), it then behooves the researchers to delineate the distinct neurodegenerative processes between SCD and anxiety, as well as the clinicians to not dismiss one over the other when patients present with both SCD and anxiety. Instead, the concurrent presence of both SCD and anxiety can then be useful to indicate a very high risk of neurocognitive disorders and potentially provide a window of opportunity for timely interventions to prevent cognitive decline, such as those related to risk-factor modification, physical exercise, and cognitive training [12, 13].

Using a large sample and a cohort study design, this study sought to address the above gap—by providing more conclusive evidence on whether SCD and anxiety truly have independent effects on the risk of mild cognitive impairment (MCI) and dementia and hence reflect two independent neurodegenerative processes that deserve separate attention by the scientific community.

## Method

### Study population

This study involves individuals recruited from approximately 39 Alzheimer’s Disease Centers across the USA between 2005 and August 2019, as available in the National Alzheimer’s Coordinating Center (NACC) database [14]. Majority of the participants (87.3%) visited the ADC to volunteer in research, while 12.5% visited the ADC to seek clinical evaluation and 0.1% had unknown reason for participation. On an approximately annual basis, the participants took part in standardized assessments (which included clinical history, physical examination, and detailed neuropsychological testing) to evaluate for incident MCI and dementia. The study included participants who fulfilled the following criteria at baseline: (1) aged ≥ 50 years, (2) diagnosed as having normal cognition at baseline (i.e., participants had completed diagnostic evaluations and found not to have MCI or dementia), and (3) provided information on SCD and anxiety. All contributing Alzheimer’s Disease Centers obtained informed consent from their participants, as well as received approval by their local institutional review boards.

### Measures

SCD was evaluated with a single yes/no question based on whether the participant perceived “a decline in memory relative to previously attained abilities.” The focus on the memory domain is not inconsistent with the current evidence in the literature, particularly in the recently proposed SCD framework [1], where memory concerns have been suggested to demonstrate better likelihood (than other non-memory concerns) in detecting prodromal neurocognitive disorders [3]. Anxiety symptoms were evaluated with a single question based on whether the participants have experienced “any signs of nervousness such as shortness of breath, sighing, being unable to relax, or feeling excessively tense” in the past month, with a choice of four responses: 0 = not present, 1 = mild (noticeable, but not a significant change), 2 = moderate (significant, but not a dramatic change), and 3 = severe (very marked or prominent; a dramatic change). In the primary analysis of this study, a score of > 0 was used to identify those with anxiety symptoms at baseline. In addition, alternative formulations of the anxiety symptoms (based on symptom severity, as well as consistency of symptom in the first 2 annual visits) were also tested in the subsequent sensitivity analyses and are further described in the “Statistical analyses” section.

The Mini-Mental State Examination (MMSE) [15] and Geriatric Depression Scale (GDS) [16] were also measured in this study and were included in the analyses as potential confounders. MMSE [15] is an 11-item measure of global cognitive function, focusing on the domains of orientation, memory, concentration, language, and constructional praxis. GDS [16] assesses the level of depressive symptoms over the past week using 15 yes/no questions. The responses are summed to produce a total score, with higher scores indicating higher levels of depressive symptoms.

The diagnoses of MCI and dementia were made based on all available information from standardized assessments [14], with 71.8% made via consensus conference and the remainder made by single clinicians. MCI was diagnosed using the modified Petersen criteria [17]. Dementia was diagnosed using the McKhann (1984) criteria [18], DSM-IV (Diagnostic and Statistical Manual of Mental Disorders–Fourth Edition) criteria [19], or the McKhann (2011) criteria [20], with further classification into the primary etiologies of Alzheimer’s dementia [18, 20], vascular dementia [21], dementia with Lewy bodies [22–24], frontotemporal lobar degeneration [23, 25–30], and other etiologies.

### Statistical analyses

Cox proportional hazard regression was conducted to evaluate the risk of MCI and dementia. Baseline presence of SCD and anxiety were concurrently included in the Cox regression to evaluate the unique risks that were attributable to each of them (after adjusting for the effects of each other). Time-to-event was defined as the duration from the baseline visit to the diagnosis of either MCI or dementia. The Cox regression adjusted for baseline covariates which may potentially confound the results, including age, sex, ethnicity, years of education, APOE e4 status, current smoking, diabetes mellitus, hypertension, hyperlipidemia, MMSE score, GDS score, history of depression, use of antidepressants, and use of anxiolytics.

The proportional hazard assumption of Cox regression was tested statistically based on whether the Schoenfeld residuals were associated with time—in the event there was significant violation of the proportional hazard assumption (*p* ≤ 0.05 in the global test on statistical significance of non-proportionality), the variables that violated the proportional hazard assumption were identified using the scaled Schoenfeld residuals and included in the Cox regression as stratified variables [3, 31, 32]. Inverse probability weighting (IPW) [33] was used in Cox regression to account for participants who did not have follow-up data. IPW is a well-accepted strategy to minimize potential bias in the results related to differential risks between those with and without follow-up data. The probabilities of being “complete cases” (those with follow-up data) were generated from logistic regression. The inverse of the probabilities were then used as weights in Cox regression, so that the results bear more semblance to those who dropped out and are less biased towards participants who provided follow-up data [3, 32–34]. Further details on IPW are available in Additional file 1.

Two sensitivity analyses were conducted to evaluate the robustness of the results when some parts of the Cox regression were modified:

1. The first sensitivity analysis examined whether the severity of anxiety symptoms can affect the risks of MCI and dementia. In this analysis, anxiety symptoms were included as an ordinal variable based on the *severity of symptoms*: 0 = not present, 1 = mild (noticeable, but not a significant change), 2 = moderate (significant, but not a dramatic change), and 3 = severe (very marked or prominent; a dramatic change).
2. The second sensitivity analysis examined whether the consistency of symptoms (over year 1 and year 2) can affect the risks of MCI and dementia. This analysis was conducted in the subset of participants with normal cognition at year 1 and year 2 (the timeline of this analysis is further depicted in Additional file 2)—those who reported anxiety or SCD at both years were deemed as having “consistent” symptoms, while those who reported anxiety or SCD at either year only were deemed as having “inconsistent” symptoms. The use of two consecutive annual visits (year 1 and year 2) to determine symptom consistency is not inconsistent with what has been done in the literature [35–37]. In particular, “consistent” SCD has also been shown to be more predictive of neurocognitive disorders in recent literature [35, 38, 39].

Additionally, a stratified analysis was conducted to evaluate the risks of MCI and dementia across different combinations of presentation, as classified by the presence of SCD or anxiety at baseline. All analyses were conducted in Stata (version 14).

## Results

The total sample size was 14,066, with a median age of 71 (interquartile range, IQR 65–77) and a median education of 16 years (IQR 14–18). Additional file 3 presents the flow diagram related to participant selection, while Table 1 shows the participant characteristics as well as the comparison between participants who did and did not develop MCI or dementia. A quarter of the participants (24.1%) only had baseline data and did not contribute to the follow-up data, while the rest of the participants had a median duration of follow-up of 4.5 years (interquartile range 2.2–7.7 years). At baseline, 1270 (9.0%) participants reported the presence of anxiety symptoms and 3809 (27.1%) reported the presence of SCD. During the period of follow-up, 1530 (10.9%) participants developed MCI while 755 (5.4%) developed dementia (with 560 being Alzheimer’s dementia, 33 vascular dementia, 48 mixed Alzheimer’s/vascular dementia, 41 dementia with Lewy bodies, 18 frontotemporal lobar degeneration, and 55 due to other or unknown etiology).

**Table 1:** Demographic information of the study participants at baseline (*n* = 14,066) and comparison between those did and did not develop dementia during the follow-up period

Variable	Overall sample (*n* = 14,066)	Participants who did not develop MCI or dementia (*n* = 11,781)	Participants who developed MCI or dementia (*n* = 2285)	*p* value^a^
Age, median (IQR)	71 (65–77)	70 (65–76)	76 (70–82)	**< 0.001**
Years of education, median (IQR)	16 (14–18)	16 (14–18)	16 (13–18)	**< 0.001**
Male sex, *n* (%)	4852 (34.5)	3956 (33.6)	896 (39.2)	**< 0.001**
Ethnicity, *n* (%)				**< 0.001**
White	11,105 (78.9)	9225 (78.3)	1880 (82.3)	
African American	1967 (14.0)	1680 (14.3)	287 (12.6)	
Others/unknown	994 (7.1)	876 (7.4)	118 (5.2)	
APOE e4 carrier, *n* (%)	3240 (23.0)	2537 (21.5)	703 (30.8)	**< 0.001**
Current smoker, *n* (%)	676 (4.8)	560 (4.8)	116 (5.1)	0.510
Diabetes mellitus, *n* (%)	1666 (11.8)	1399 (11.9)	267 (11.7)	0.800
Hypertension, *n* (%)	6781 (48.2)	5568 (47.3)	1213 (53.1)	**< 0.001**
Hyperlipidemia, *n* (%)	6888 (49.0)	5751 (48.8)	1137 (49.8)	0.410
MMSE score, median (IQR)	29 (28–30)	29 (29–30)	29 (28–30)	**< 0.001**
GDS score, median (IQR)	1 (0–2)	1 (0–2)	1 (0–2)	**< 0.001**
History of depression, *n* (%)	3720 (26.4)	3107 (26.4)	613 (26.8)	0.650
Use of antidepressants, *n* (%)	2666 (19.0)	2227 (18.9)	439 (19.2)	0.730
Use of anxiolytics, *n* (%)	1659 (11.8)	1410 (12.0)	249 (10.9)	0.150
Presence of anxiety symptoms, *n* (%)	1270 (9.0)	1017 (8.6)	253 (11.1)	**< 0.001**
Presence of SCD, *n* (%)	3809 (27.1)	2969 (25.2)	840 (36.8)	**< 0.001**

*MCI* mild cognitive impairment, *IQR* interquartile range, *MMSE* Mini-Mental State Examination, *GDS* Geriatric Depression Scale, *SCD* subjective cognitive decline

^a^Test of difference between participants with and without longitudinal follow-up data: chi-square test for categorical variables, and Mann-Whitney *U* test for continuous variables. Bold-faced *p* values are ≤ 0.05

In Cox regression, both anxiety symptoms and SCD demonstrated independent risks of MCI and dementia, with a HR of 1.3 for anxiety and 1.9 for SCD (Table 2). The findings remained robust in the two sensitivity analyses (Table 3), with both anxiety and SCD consistently demonstrating their independent effects. In the first sensitivity analysis, anxiety was included in Cox regression as an ordinal variable based on the severity of symptoms (i.e., mild, moderate, or severe)—mild and moderate symptoms had similar risk estimates (HR 1.3–1.4), while severe symptoms had a relatively higher risk estimate (HR 2.3). In the second sensitivity analysis, anxiety and SCD were included in Cox regression based on the consistency of the symptoms in the first 2 annual visits of the study (i.e., those who reported the presence of symptoms at year 1 and year 2 were deemed to have “consistent” symptoms, while those who reported the symptoms only at year 1 *or* year 2 were deemed to have “inconsistent” symptoms)—the risk estimates of anxiety remained similar regardless of whether the symptoms were consistently reported in the first 2 years. In contrast, the risk estimate of consistent SCD (HR 2.5) was higher than that of inconsistent SCD (HR 1.6).

**Table 2:** The risk of mild cognitive impairment and dementia based on the presence of anxiety and subjective cognitive decline at baseline (*n* = 14,066)

Presence of symptoms	No. of MCI and dementia/total (%)	Model 1 (unadjusted)^a^		Model 2^b^		Model 3^c^		Model 4 (final)^d^	
		HR (95% CI)	*p*	HR (95% CI)	*p*	HR (95% CI)	*p*	HR (95% CI)	*p*
Anxiety									
No	2032/12796 (15.9)	1.0 (Ref)	Ref	1.0 (Ref)	Ref	1.0 (Ref)	Ref	1.0 (Ref)	Ref
Yes	253/1270 (19.9)	1.4 (1.2–1.6)	< 0.001	1.6 (1.4–1.8)	< 0.001	1.5 (1.3–1.7)	< 0.001	1.3 (1.2–1.5)	< 0.001
SCD									
No	1445/10257 (14.1)	1.0 (Ref)	Ref	1.0 (Ref)	Ref	1.0 (Ref)	Ref	1.0 (Ref)	Ref
Yes	840/3809 (22.1)	2.0 (1.8–2.2)	< 0.00	2.0 (1.9–2.2)	< 0.001	2.0 (1.8–2.2)	< 0.001	1.9 (1.7–2.1)	< 0.001

*SCD* subjective cognitive decline, *HR* hazard ratio, *CI* confidence interval, *Ref* reference group

^a^Cox regression included only anxiety and SCD without covariate adjustment

^b^Cox regression adjusted for covariates of age, sex, and ethnicity

^c^Covariate adjustment as in model 2, with additional adjustment for years of education, APOE e4 status, current smoking, diabetes mellitus, hypertension, hyperlipidemia, and Mini-Mental State Examination score

^d^Covariate adjustment as in model 3, with additional adjustment for total score on Geriatric Depression Scale, history of depression, use of antidepressants, and use of anxiolytics

**Table 3:** Results from the two sensitivity analyses to evaluate the robustness of the main findings

Analyses	No. of MCI and dementia / Total (%)	Hazard ratio (95% CI)^a^	*p* value
Sensitivity analysis 1: severity of anxiety (*n* = 14,066)			
Anxiety^b^			
No symptoms	2032/12796 (15.9)	1.0 (Ref)	Ref
Mild symptoms	182/908 (20.0)	1.3 (1.1–1.5)	0.004
Moderate symptoms	55/311 (17.7)	1.4 (1.0–1.9)	0.032
Severe symptoms	16/51 (31.4)	2.3 (1.3–4.1)	0.004
SCD			
No	1445/10257 (14.1)	1.0 (Ref)	Ref
Yes	840/3809 (22.1)	1.8 (1.7–2.0)	< 0.001
Sensitivity analysis 2: consistency of symptoms in the first 2 years of the study (*n* = 6926)			
Anxiety^c^			
No anxiety	707/5982 (11.8)	1.0 (Ref)	Ref
Inconsistent anxiety	130/762 (17.1)	1.6 (1.3–1.9)	< 0.001
Consistent anxiety	34/182 (18.7)	1.7 (1.1–2.6)	0.013
SCD^c^			
No SCD	456/4630 (9.9)	1.0 (Ref)	Ref
Inconsistent SCD	237/1459 (16.2)	1.6 (1.3–1.9)	< 0.001
Consistent SCD	178/837 (21.3)	2.5 (2.0–3.0)	< 0.001

*SCD* subjective cognitive decline, *CI* confidence interval, *Ref* reference group

^a^Model adjusted for age, sex, ethnicity, years of education, APOE e4 status, current smoking, hypertension, hyperlipidemia, diabetes mellitus, Mini-Mental State Examination score, total score on Geriatric Depression Scale, history of depression, use of antidepressants, and use of anxiolytics

^b^Anxiety symptoms were included in the analysis as an ordinal variable, based on the severity of symptoms: 0 = not present, 1 = mild (noticeable, but not a significant change), 2 = moderate (significant, but not a dramatic change), and 3 = severe (very marked or prominent; a dramatic change)

^c^This analysis was conducted in the subset of participants with normal cognition at year 1 and year 2—those who reported anxiety or SCD at both years were deemed as having “consistent” symptoms, while those who reported anxiety or SCD at either year only were deemed as having “inconsistent” symptoms

**Fig. 2:**
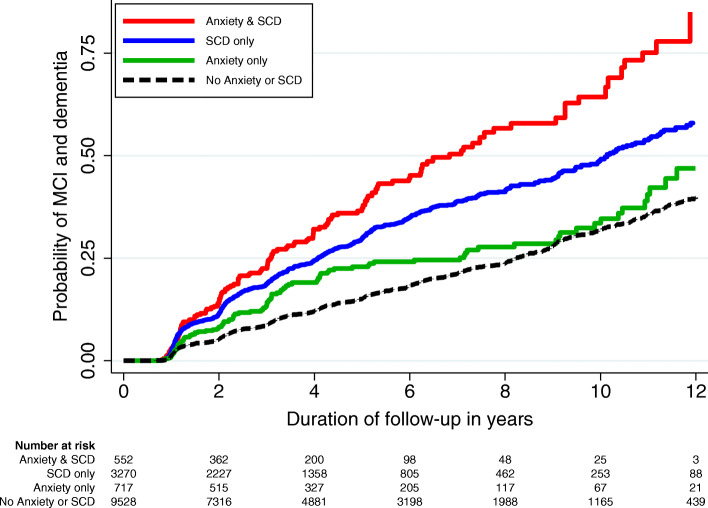
Kaplan-Meier curves reflecting the risk of mild cognitive impairment (MCI) and dementia, based on the presence of anxiety symptoms and subjective cognitive decline at baseline (*n* = 14,066)

The risks of MCI and dementia were further evaluated across different combinations of presentation, classified by the presence of anxiety or SCD at baseline. As shown in Table 4, the HR of MCI and dementia increased incrementally from anxiety only, to SCD only, and to both anxiety and SCD. Individuals without anxiety or SCD had a 25% probability of developing MCI or dementia by 8.2 years of follow-up. This duration became as short as 3.1 years in the presence of both anxiety and SCD. The differential risks across the various combinations of presentation are further visible in the Kaplan-Meier curve in Fig. 2.

**Table 4:** Risk of mild cognitive impairment and dementia associated with the different combinations of presentation, based on the presence of anxiety symptoms and subjective cognitive decline at baseline (*n* = 14,066)

Different combinations of presentation	No. of MCI and dementia/total (%)	Hazard ratio (95% CI)^a^	*p* value	Survival (25th centile) in years (95% CI)^b^
No anxiety or SCD	1332/9535 (14.0)	1.0 (Ref)	Ref	8.2 (7.9–8.6)
Anxiety only	113/722 (15.7)	1.4 (1.1–1.8)	0.002	7.1 (4.2–9.9)
SCD only	700/3261 (21.5)	1.9 (1.7–2.1)	< 0.001	4.1 (3.7–4.4)
Both anxiety and SCD	140/548 (25.6)	2.4 (1.9–2.9)	< 0.001	3.1 (2.4–3.7)

*CI* confidence interval, *MCI* mild cognitive impairment, *SCD* subjective cognitive decline, *Ref* reference group

^a^Model adjusted for baseline variables of age, sex, ethnicity, years of education, APOE e4 status, current smoking, hypertension, hyperlipidemia, diabetes mellitus, Mini-Mental State Examination score, total score on Geriatric Depression Scale, history of depression, use of antidepressants, and use of anxiolytics

^b^The estimated time that is needed for a quarter of the participants to develop MCI or dementia. The 95% CI was computed with 1000 bootstrap sampling

## Discussion

This study examined the relationship between two correlated constructs—anxiety symptoms and SCD—and provided more conclusive evidence on the independent effects of anxiety and SCD on the subsequent development of neurocognitive disorders, based on a large sample of 14,066 participants, after adjusting for the mutual effects of each other, as well as after accounting for a wide range of key confounders. The risk of MCI and dementia was independently present in both anxiety and SCD (HR of 1.3 and 1.9, respectively), with the risk being highest among those who endorsed both anxiety and SCD (HR 2.4)—participants with co-occurring anxiety and SCD had a 25% probability of developing MCI or dementia by 3.1 years (in contrast to 8.2 years among those without anxiety or SCD). The results remained robust in the sensitivity analyses, even after the severity and consistency of symptoms were accounted for in the analyses. In particular, the sensitivity analyses revealed that higher severity of anxiety was associated with higher risk of MCI and dementia, but consistency of anxiety symptoms over the first 2 years had minimal impact on the risk estimates (i.e., the risks were similar regardless of whether anxiety was reported at one time point only or consistently over the first 2 years).

Inasmuch as the literature has reported the strong correlation between SCD and anxiety symptoms [2], this study demonstrated that the two likely represent distinct constructs which independently predict the risk of neurocognitive disorders, consistent with the postulated relationship in Fig. 1c. The findings can have both clinical and research implications. From the *clinical perspective*, the findings highlight the relevance of both constructs to health services that are involved in the care of older persons, and that clinicians should not dismiss one over the other when patients present with both anxiety and SCD. For example, among older patients with anxiety disorders, the additional complaints of SCD should not be dismissed as merely being part of “anxiety symptoms” (which invariably shifts the focus of care to the alleviation of this “anxiety symptom” using psychiatric medications). Vice versa, among older patients with known SCD, the additional complaints of anxiety symptoms should also not be dismissed as merely a psychological reaction to their concerns about the SCD. On the contrary, anxiety and SCD should be seen as *independent* predictors of neurocognitive disorders, and the co-occurrence of both should alert clinicians to the much higher risk of neurocognitive disorders, which may then prompt more intensive interventions to prevent cognitive decline (such as those related to risk-factor modification, physical exercise, and cognitive training) [12, 13], enrolment into preventive trials for dementia [3, 32], and closer monitoring of cognitive function over time to allow timely diagnosis of cognitive impairment [40–43].

From the *research perspective*, the findings may possibly indicate some differences in the underlying neurodegenerative processes between SCD and anxiety—the two constructs may either reflect the involvement of two distinct neurobiological pathways that lead to neurocognitive disorders, or a common neuropathology that has affected two distinct anatomical regions in the brain [3]. Prior studies have already implicated different sets of neurobiology for SCD and anxiety. For example, SCD has been linked to the cholinergic system in the basal forebrain [44], as well as to white matter lesions, smaller left hippocampal volumes, and temporal lobe atrophy [45]. In contrast, anxiety has been linked to lower metabolism in the bilateral entorhinal cortex, anterior parahippocampal gyrus, and left superior temporal gyrus and insula, as well as a relatively preserved amygdala volume [46]. Future research is needed to further delineate the similarities and differences in the neurobiology between these two distinct constructs, which may potentially enrich our understanding on the pathogenesis of neurocognitive disorders as well as identify novel drug targets to inform the development of disease-modifying drugs for dementia [3].

Several limitations should be considered. First, the participants in the study involved those who volunteered at the Alzheimer’s Disease Centers. They may be more representative of patients who voluntarily present to healthcare settings than those in the community. Second, 22.1% of the participants did not provide follow-up data. Such missing data were addressed using IPW [33] to minimize any potential bias. Third, single yes/no questions were used to identify the exposures-of-interest in this study (SCD and anxiety). Although they should not invalidate the findings of this study, single questions may not be as sensitive as multi-item questionnaires in identifying SCD and anxiety. In particular, the single question that captures SCD focused on the memory domain—it may not have captured the other non-memory domains [47]. Fourth, the primary analysis in this study was based on one-time assessments of anxiety and SCD (i.e., at baseline). Arguably, the symptoms of anxiety and SCD can fluctuate over time, and hence, measurements at one time point may not necessarily be a true reflection of participants’ anxiety or concerns about SCD. This limitation is addressed in the second sensitivity analysis of the study, with the results remaining robust even after the consistency of symptoms (in the first 2 years) was accounted for in the analysis. Fifth, the diagnoses of MCI and dementia were made by single clinicians in 28.2% of the participants. They may not necessarily be as accurate as those made via consensus conference.

## Conclusion

Among older persons with normal cognition, SCD and anxiety symptoms are independently associated with the risk of neurocognitive disorders, with the risk further compounded when SCD and anxiety symptoms co-occur. The findings highlight the potential usefulness of the two constructs to identify high-risk populations for preventive interventions and trials, as well as point to the need for further research to clarify on the neurobiological distinctions between the two constructs to enrich our understanding of neurocognitive disorders.

## Supplementary information

**Additional file 1.** Details on the conduct of inverse probability weighting to account for those who did not have follow-up data after the first visit.

**Additional file 2.** Time line of the second sensitivity analysis.

**Additional file 3.** Participant enrolment and exclusion details.

## Data Availability

The data were obtained from the National Alzheimer’s Coordinating Center (NACC). For further information on access to the database, please contact NACC (contact details can be found at https://www.alz.washington.edu/WEB/researcher_home.html).

## Abbreviations

CI: Confidence interval
GDS: Geriatric Depression Scale
HR: Hazard ratio
IQR: Interquartile range
IPW: Inverse probability weighting
MCI: Mild cognitive impairment
MMSE: Mini-Mental State Examination
NACC: National Alzheimer’s Coordinating Center
NIA-AA: National Institute on Aging–Alzheimer’s Association
SCD: Subjective cognitive decline
