# Supplementary material for: Subjective cognitive decline, anxiety symptoms, and the risk of mild cognitive impairment and dementia
Source: Alzheimers Res Ther. 2020 Sep 11;12:107. doi: 10.1186/s13195-020-00673-8 (PMC7488541; doi:10.1186/s13195-020-00673-8)
Supplement: Supplementary file 1 — Additional file 1. Details on the conduct of inverse probability weighting to account for those who did not have follow-up data after the first visit. [file 13195_2020_673_MOESM1_ESM.docx]

**Additional file 1.** Details on the conduct of inverse probability weighting to account for those who did not have follow-up data after the first visit.

In inverse probability weighting, the “complete cases” (those with follow-up data, n=10,671) were weighted in cox regression by the inverse of their probability of being a complete case so that the results bear more semblance to those who did not contribute to follow-up data (n=3,395). The probability of being a complete case was generated from logistic regression, with the predictors based on the variables included in the primary analysis (presence of anxiety, presence of subjective cognitive decline, age, sex, ethnicity, years of education, APOE e4 status, current smoking, hypertension, hyperlipidemia, diabetes mellitus, Mini-Mental State Examination score, total score on Geriatric Depression Scale, history of depression, use of antidepressants, and use of anxiolytics) as well as other auxiliary variables that may help to predict drop-out, including marital status (Married/Widowed/Separated/Single/Other), living arrangement (Alone/Spouse/Relative/Group), type of residence (Private residence/Retirement community/Assisted living), primary reason of participation (Research/Clinical evaluation/Research and clinical evaluation), and primary source of referral (Healthcare providers/Non-professional contact/Other). This logistic model had an acceptable fit in the Hosmer-Lemeshow test (evidenced by the non-significant p-value of 0.092), with the calibration plot showing agreement between the predicted probability and the observed frequency as shown below:
