# Supplementary material for: Subjective cognitive decline, anxiety symptoms, and the risk of mild cognitive impairment and dementia
Source: Alzheimers Res Ther. 2020 Sep 11;12:107. doi: 10.1186/s13195-020-00673-8 (PMC7488541; doi:10.1186/s13195-020-00673-8)
Supplement: Supplementary file 2 — Additional file 2. Time line of the second sensitivity analysis. [file 13195_2020_673_MOESM2_ESM.docx]

**Additional file 2.** Time line of the second sensitivity analysis.

MCI, mild cognitive impairment.

.
