# Supplementary material for: Subjective cognitive decline, anxiety symptoms, and the risk of mild cognitive impairment and dementia
Source: Alzheimers Res Ther. 2020 Sep 11;12:107. doi: 10.1186/s13195-020-00673-8 (PMC7488541; doi:10.1186/s13195-020-00673-8)
Supplement: Supplementary file 3 — Additional file 3. Participant enrolment and exclusion details. [file 13195_2020_673_MOESM3_ESM.docx]

**Additional File 3.**  Participant enrolment and exclusion details.

NACC, National Alzheimer’s Coordinating Center; MCI, mild cognitive impairment; NC, normal cognition; SCD, subjective cognitive decline.

Baseline diagnosis of NC

n = 15,331

No data on anxiety and SCD at baseline

n = 776

Available data on anxiety and SCD at baseline

n = 14,555

Age <50 years

n = 489

Age ≥50 years

n = 14,066

Baseline diagnosis of MCI or dementia

n = 19,952

Available data on covariates

n = 35,283

Included in current study

n = 14,066

No data on covariates

n = 4,308

Participants in NACC database

n = 39,591
